# How do adolescents navigate COVID-19 information, and why does it matter?

**DOI:** 10.7189/jogh.11.03110

**Published:** 2021-12-25

**Authors:** Astha Ramaiya, Pablo Villalobos, Effie Chipeta, Jairo Vanegas Lopez, Matilde Maddaleno, Xiayun Zuo, Eric Mafuta, Aimee Lulebo, Jakevia Green, Lisa Richardson, Kristin Mmari

**Affiliations:** 1 Department of Population, Family and Reproductive Health, Bloomberg School of Public Health, Johns Hopkins University, Baltimore, Maryland, USA; 2 University of Santiago, Santiago, Chile; 3 Centre for Reproductive Health, College of Medicine, Blantyre, Malawi; 4 NHC Key Lab of Reproduction Regulation (Shanghai Institute for Biomedical and Pharmaceutical Technologies), Fudan University, Shanghai, China; 5 Kinshasa School of Public Health and College of Medicine, Kinshasa, Democratic Republic of Congo; 6 Institute of Women and Ethnic Studies, New Orleans, Luisiana, USA

The world has not seen a global health crisis as devastating as COVID-19 since the 1918 flu pandemic. While adults are more likely to become physically ill, COVID-19 has profound impacts on adolescents’ mental health and emotional well-being [1,2]. According to the WHO, adolescents are defined as those who are ages 10 to 19 [3]. During COVID-19, social distancing measures and school closures have not only led to significant educational disruptions but have also limited important opportunities for peer social connection, identity development, and independence [4,5].

Paramount to this global pandemic has also been the dissemination and use of accurate and reliable COVID-19 information to help curtail the spread of the virus [6-8]. From previous health emergencies and pandemics, we know that health information needs to be succinct, honest, valid and verifiable [6,9]. A systematic review during the 2009 H1N1 pandemic showed that for people to follow preventative practices they had to have: 1) trust in public officials and the source of information, 2) increased perceived severity about the disease, 3) knowledge about the disease, and 4) greater media exposure [10]. In the current pandemic, people have been using social media and networks to obtain information about COVID-19, which means that the information may not always be accurate. This produces negative effects on the official national and international information. Without including the “bubble filters”, the algorithm associates the preference of a user and unites it with that of other similar users, producing a loop of similar content preventing the user from seeing other different sources to assess the validity of the claim [11]. This affects the possible dissemination of erroneous, alarmist, and exaggerated information that can cause fear, stress, depression, and anxiety in both healthy and sick people [11].

Yet, amid the COVID-19 pandemic, the number of different mediums and platforms for delivering accurate health information has been overwhelming [6-8]. For adolescents who are trying to navigate how to stay safe, protect their loved ones, and cope with an uncertain future, understanding what sources of information to trust can be stressful in and of itself [12-14]. Yet, there is limited information about how adolescents receive accurate information about COVID-19, and how they decide what is or is not trustworthy. Since COVID-19 is a global disease, it is also not clear whether adolescents’ sources of COVID-19 information vary by culture or even by gender. This information is critical if we are to prevent the spread of the virus by practicing preventative behaviors, help populations cope with uncertainty and fear, and promote behavior change at the individual and community levels to make these behaviors a habit and prevent future outbreaks [6].

**Figure:**
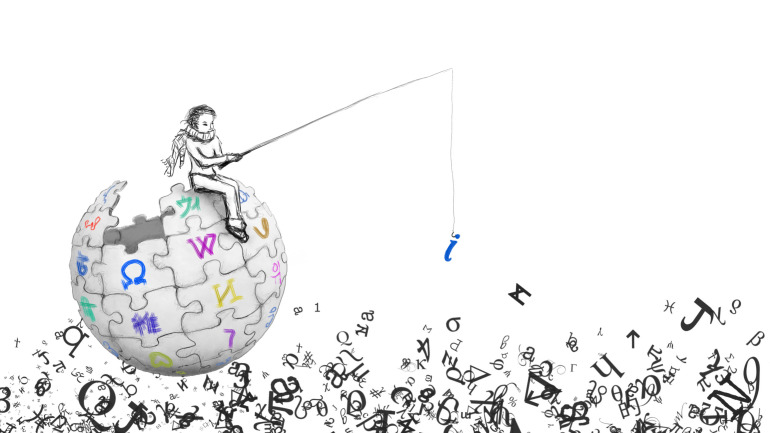
Photo: By Helixitta; Wikimedia Commons.

To fill in this knowledge gap, we utilized data from the Global Early Adolescent Study, a longitudinal study conducted in ten countries across five continents among adolescents living in urban poor settings [15]. In 2020, a sub-study of GEAS was awarded to examine how adolescents were experiencing the pandemic across different cultural settings. We use the *Know, Feel, Do Framework* to understand the qualitative data from this sub-study and deduce the following objectives [16]: 1) To identify the main sources of COVID-19 information among boys and girls across sites and how that contributes to knowledge about the virus; 2) To determine how adolescents feel about the sources they are receiving information from and 3) To explore the ways in which adolescents determine the trustworthiness of COVID-19 information sources. For all three objectives, we compared between sites and gender.

This study uses focus group discussion (FGD) data from urban poor communities in Kinshasa (Democratic Republic of Congo (DRC)), Shanghai (China), Semarang and Denpasar (Indonesia), New Orleans (USA), and Flanders (Belgium). In all sites except one, the sampling frame was current GEAS participants, who were already part of the longitudinal study. In Shanghai, the younger group (14-15 years) were GEAS participants and older (16-18 years) adolescents were from a nearby high school since the older GEAS participants had changed schools across the city. Adolescents were eligible to participate in the FGDs if they were 14-18 years. The interviews were conducted between June and November 2020.

All sites followed a common protocol and used the same FGD guides (Table S1 in the **Online Supplementary Document**), but sampling and data collection procedures varied by sites. Table S2 in the **Online Supplementary Document** shows how sampling, recruitment and data collection occurred. Data collection for the FGDs occurred in schools, community centers or online. In Kinshasa and Shanghai, since the COVID-19 cases were relatively low during data collection, the FGDs were conducted in-person with all COVID-19 precautions and national or local regulations. In Semarang, Denpasar, New Orleans and Flanders the FGDs were conducted online using the Zoom videoconferencing platform.

A total of 4 FGDs were conducted in almost all sites, with the exception of Flanders, which only conducted two mixed-sex FGDs. Each FGD in all sites had between 6 and 10 participants. Table S3 in the **Online Supplementary Document** shows that stratification differed based on site.

Trained facilitators in each site conducted the interviews. All conversations were audio-recorded, transcribed and translated from the local language to English. The FGDs elicited information on knowledge, attitudes, sources of information, preventative behavior practiced, current and future impact of COVID-19 and types of support needed. For this study, we focused on the questions which asked about sources of information (including how they first heard about COVID-19), which sources were considered trustworthy/untrustworthy (and how trustworthiness was determined), knowledge about COVID-19, and attitudes about the virus. Table S4 in the **Online Supplementary Document** outlines the different codes by site.

All partner sites applied for and received ethical approval from their local ethical boards. Kinshasa received ethical approval from Kinshasa School of Public Health; Shanghai received approval from Shanghai Institute of Planned Parenthood Research; Indonesia received approval from Universitas Gadjah Mada in Indonesia; New Orleans received approval from Institute of Women and Ethnic Studies; and Flanders received approval from University of Ghent. The study was deemed exempt for secondary data collection by the Johns Hopkins Bloomberg School of Public Health’s Institutional Review Board.

We used an inductive thematic analytical approach to code and analyze emerging themes across different cultural contexts and assess both similarities and differences based on the study’s objectives [17].

Two coders initially coded the first four transcripts to check for inter-rater reliability. Thereafter, the two coders independently coded the other 18 transcripts. Once the coding was completed, individual country and cross-site matrices were created for each code based on stratification. All analyses were conducted on Atlas.ti 9.1 [18]. Key codes for this manuscript are outlined in the **Online Supplementary Document**.

## WHAT ARE THE DIFFERENT SOURCES OF INFORMATION ABOUT COVID-19, AND HOW DO THEY CONTRIBUTE TOWARD KNOWLEDGE?

### Sources of information and type of information

When adolescents were asked about the sources of information for COVID-19, the most frequently-cited sources included news reports and official government reports through television, radio and social media, as well as discussions with family members/neighbors, health professionals and teachers:

“CCTV’s news channel and official reports such as Toutiao and so on.” *(Shanghai older boys)*

“From the news and my parents*” (Semarang higher SES girl)*

In Shanghai, boys and girls equally reported that they received their information about COVID-19 from official government sources on television and the internet:

“Official website [governmental website]. It is the official, and information that the national governments released.” *(Shanghai older girls)*

In Indonesia and Kinshasa, however, boys discussed receiving COVID-19 information from health professionals and reputable health organizations (such as WHO), while girls discussed obtaining information about COVID-19 from family members and television:

“Reading from the government news, from WHO” *(Denpasar higher SES boy)*

“From TV and parents‬‬‬‬” *(Semarang higher SES girl)‬*

The information they received from these sources included virus description, transmission, symptoms, prevention and origin of the virus.

### Description of the virus

Participants in all sites provided a description of the virus in terms of its severity and spread. Both boys and girls mentioned the virus was fatal and that it had spread across the world:

“Coronavirus is a very contagious disease. When we talk about the pandemic we saw that it concerns the whole world‬…TV, radio ‬[through The Minister of Health and the Chief of State]” *(Kinshasa boy)*

*“But usually it got worse and there were more deaths… But yes, because our school sent us a lot of messages about it on smart school [online learning platform], and kept us very well informed about everything*.” *(Flanders, mixed)*

### Transmission

Most participants understood that COVID-19 is spread through respiratory molecules in the air through coughing and sneezing. There was the common belief that the virus spreads through touching things on surfaces or interacting with those who are infected:

“Well, the transmission is mainly through the mucous membranes, such as the mucous membranes of mouth or eyelids. If mucous membranes contacted with the virus, he or she will be infected … Watch the news, through mobile phone.‬” *(Shanghai younger girl)*

“By greetings, hugs and also coughs… TV, radio‬” *(Kinshasa boy)*

However, there were some transmission modes mentioned by adolescents that seemed to be related to the beliefs regarding the origin of the virus. Girls in Shanghai, Indonesia, and Kinshasa said it could be transmitted by eating wild animals and uncooked food:

*“*Eat meals that are really cooked well. Like if it’s a chicken it can’t be cooked medium-well or rare.‬‬‬‬..Uhh from social media. And from TV there are a lot of people lying around in the place. And for example the ladies around the house usually talk about it at home (laughs)” *(Denpasar higher SES girl)*

*“*I think we should try not to eat wild unknown creatures…From the notice board in the community.” *(Shanghai younger girl)*

### Symptoms

Overall, across all the sites, there was a high level of knowledge about COVID symptoms. Participants mentioned fever, sweating, coughing, sore throat, loss of taste, vomiting, body aches and headaches:

“It’s almost like the flu, but it's worse.‬ Dry cough and fever.‬ Yes. Vomiting. Shortness of breath. Oh, chills...taste... On CNN… On most news channels.” *(New Orleans lower SES girls)*

Adolescents in Kinshasa were in a unique situation where the COVID-19 pandemic converged with the Ebola epidemic. This could explain some incorrect knowledge about COVID-19 symptoms (vomiting blood, making holes) at the site:

“*Sweating and coughing‬; Respiratory difficulty and excessive fever a cold; It manifests itself by fever and cough, when you are afflicted you will start to vomit blood… I learned about it through social media and through the covid awareness campaign that was ‬being done‬‬” (Kinshasa boys)*

“Us, we saw on the internet that it made little holes in the people affected by Covid-19. On the image it was even a little girl who had holes all over her body.‬” *(Kinshasa girl)*

### Prevention

Across all sites, participants discussed wearing masks, social distancing, staying home and washing hands as preventative behaviors they heard from various sources of information:

“The main preventive measures including not standing too close when communicating with others, taking good personal hygiene protection, and wearing a mask… I got this knowledge mainly through the Central News, and I also found it by searching on the Internet‬” (*Shanghai older boy)‬‬*

Girls in comparison to boys (in Indonesia and Kinshasa) mentioned alternative natural remedies to prevent COVID-19 such as exercising, eating fruits/vegetables, taking steam bath, sunbathing to get Vitamin D, eating Vitamin C and covering self in eucalyptus trees to prevent COVID-19. The source of this information for these natural remedies were parents, teachers or neighbors:

“Wearing a mask when going outside, then keeping our distance from other people and maintain the body immune system… From my parents, because they told me to sunbathe in the morning‬‬‬” *(Semarang higher SES girl)*

“For us, it was a neighbour who had come by the house to tell Mom‬ to buy the kongo bololo [traditional medicinal plant] for us. She said that this is what protects against‬ corona virus. And I assure you that we took a good amount of it almost‬ every day.‬” *(Kinshasa girl)*

### Origin of virus

Across all sites, participants mentioned that the geographical origin of the virus was China:

“I know it first from Instagram, uhh there were a lot of accounts who posted about it, about covid 19, uh not yet... they posted about corona first... it was still called corona virus back then. And then... it's going to be a pandemic, so on Instagram there were quite a lot who made jokes about the new Chinese virus” *(Denpasar higher SES boy)*

Participants in Shanghai and Kinshasa also mentioned the origin of the virus was from eating animals:

*“*The first time I learned about the 2019-nCoV was through a mobile browser. I understood that there was a new disease caused by someone eating bats in Wuhan. At first, I thought it was a small disease caused by eating animals*.” (Shanghai older boy)*

However, older girls in Shanghai mentioned that the geographical origin of the virus was in western countries and boys in Kinshasa outlined that God was upset at humankind (especially in western countries) for practicing homosexuality and sodomy:

*“*I knew COVID-19 in December. In December, I learned that more than 2000 people in the United States were infected with influenza through some channels. Then I began to pay attention to the information about this aspect [geographical origin]. In January, I heard that a large amount of people affected in Wuhan… Bilibili, Weibo, and the Dingxiang doctor on Wechat, as well as the real-time record. Now I only focus on the information posted by the account number of the Shanghai Communist Youth League on Bilibili.‬ *‬*” *(Shanghai older girl)*

“I immediately believed it was real because ever since I started following the news that was all they talked about and when I learned it arrived here, I thought that was true‬…I learned that it was all because of evil practices of sodomie and homosexuality and that it is God who is angry with mankind, especially in western countries‬” *(Kinshasa boy)*

## HOW DO ADOLESCENTS FEEL ABOUT THE SOURCES OF INFORMATION, AND HOW DO THESE PERCEPTIONS VARY BY SITE AND GENDER?

### Perceived severity of virus

Participants in almost all sites (except Shanghai), talked about an increase in perceived severity in relation to when they heard about the virus and when it reached their country. This was discussed to a greater extent by boys in comparison to girls:

“For me, I first heard at school that there was a new virus and that people, especially in China, suffered a lot from it, but I never thought that it would come to Europe and that it would be so big that the whole world would suffer from it so it was quite a shock when it also came to Belgium and so many people got infected.*” (Flanders, mixed)*

*“*We learned more than 600 people died every day in foreign countries and that was scary. That was what disciplined me to get used to wearing the mask… I learned that from the television and the Top congo radio*‬” (Kinshasa, boy)*

Most participants in Shanghai noted a low perceived severity, because during the four-month school lockdown, the number of new cases and mortality due to COVID-19 was low in the city. Adolescents stated they believed in their government’s controlling measures and their self-efficacy to practice the preventative measures:

“I felt a little worried when the epidemic began, for example, whether the disease would be highly infectious or would it have a great impact on my life. But I felt that the government's controlling measures and its effects were OK, so I thought it was not so bad.” (Shanghai older boy)

“Yes, but it should not be as serious as this time. After all, we know how to protect ourselves, for example, the outbreak of epidemic in Wuhan were so serious, while that (the controlling of) this spread in Beijing recently was much better.*” (Shanghai older girl)*

Interestingly, participants from Flanders and boys from Denpasar mentioned they stopped watching the news since it increased their anxiety and stress or because the pandemic was being hyped up:

“For me it was… in the beginning when we were in lockdown, that was also very much,… yes, I have watched the news and I always wanted to know a lot about it, so I watched the news every night. But usually it got worse and there were more deaths. And I noticed that I got a lot of stress from that so then I, yes, didn't watch the news that much anymore ‬‬‬‬‬” *(Flanders mixed)*

“In my opinion, this covid pandemic can be over, if a lot of people start to... if everyone start to follow the protocol and... what's it called... and uhhh and... back pedall from sharing the news that worries others you know, we're supposed to share the news that lift our spirits up, so that the pandemic can be over, you know.*” (Denpasar higher SES boy)*

## WHAT ARE THE WAYS IN WHICH ADOLESCENTS DETERMINE THE TRUSTWORTHINESS OF COVID-19 INFORMATION SOURCES?

In general, adolescents across sites discussed government sources of information and news outlets as the most trustworthy sources:

“Radio Top congo, because they provided good information and they invited the Minister of Health on their show to explain the covid situation in our country” *(Kinshasa boy)*

“Oh yeah, I was um I have Apple news. And I watch the news a lot too.” *(New Orleans girl)*

Sources that were considered untrustworthy were social media, advertisements/screen shots on the internet, and word-of-mouth:

“Yes so with news sites, you can be sure they're telling the truth but on social media you're not sure what's true.” *(Flanders Mixed)*

“Rumours on the grapevine…Screenshots of chats, websites and other sources like these.” *(Shanghai, girl)*

Girls in Semarang and Kinshasa reported that their most trustworthy sources of COVID-19 information were either their family members or teachers, whereas boys in Denpasar discussed health professionals/health organizations as their trustworthy source:

“…the school because at school they can’t lie to us” *(Kinshasa girl)*

“The trusted source is usually the sources said by doctors or experts who handle covid 19” *(Denpasar boy)*

These sources were considered trustworthy based on a mental ranking of priorities:

1) **If the information was perceived to be logical**. Regardless of the source, if adolescents perceived the information as being logical, they were more likely to perceive it as trustworthy. This was based on the information they knew about the virus, preventative practices and how the virus is transmitted from one person to another.

“The source maybe (untrustworthy). Because travelling doesn't mean you catch covid. Because we already put a mask on, keeping our distance” *(Semarang boy)*

Girls in Denpasar and Kinshasa also stated they believe the information if they had personally experienced and/or witnessed the severity of the virus:

“And with what I myself had seen when we went to visit someone in a hospital, the way they wrapped corpses that died with COVID-19.” *(Kinshasa, girl)*

Additionally, adolescent boys and girls in all sites except Flanders found that if they could compare the information they were receiving about COVID-19 with information they received about other diseases, they were more likely to perceive it as trustworthy.

“For me, I am sure that COVID-19 will come to an end, but it will remain‬ like other diseases: Malaria, yellow fever and others. It is necessary to respect‬ cautionary measures, stand at one meter apart, wash your hands regularly‬” *(Kinshasa boy)*

*“It’s almost like the flu, but it's worse.‬”* *(New Orleans girl)*

2) **If they could cross-verify:** Adolescent girls from Semarang, Kinshasa, and Shanghai were also more likely to perceive that a source was trustworthy if they could verify the information with close family members or teachers.

“If I got it from the news on TV, I usually confirm it to my parents whether it's right or wrong, can I do it, such things” *(Semarang girl)*

Boys in Shanghai and Flanders, however, reported that they verified their sources across other internet sites:

“I got this knowledge mainly through the Central News, and I also found it by searching on the Internet, which is also credible in my mind.*” (Shanghai boy)*

Adolescent boys and girls in New Orleans and Flanders mentioned they received notifications on social media platforms alerting them if the information they were viewing on that platform was not factual, helping them to cross-verify their sources:

*“*Um, because like when you see it on the media they do like a fact check. And sometimes it's like they say this is own this is not real the real like what's really going on. And they give you the website to the CDC most of the time*.” (New Orleans girl)*

**Figure 1:**
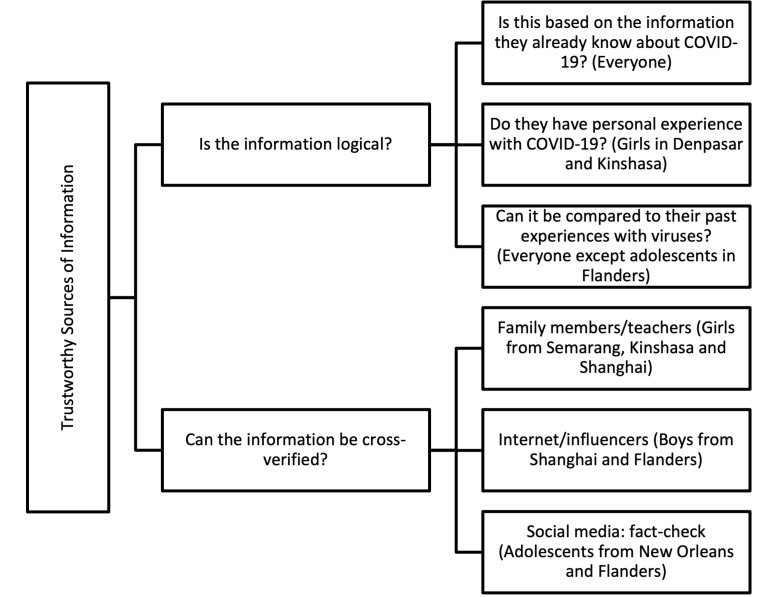
Decision making tree on how adolescents determine trustworthiness of sources.

**Figure 1** outlines how these priorities were ranked across the sites and by sex.

## IMPLICATIONS

This paper sought to identify the main sources of COVID-19 information and how adolescents process the messages from those sources to inform their own preventative behaviors about the disease. The findings show that although adolescents mentioned a variety of different sources of information, the most common sources were news reports (government or non-government), health professionals, family members, and teachers. Adolescents in Shanghai discussed trusting the government sources to a greater extent than adolescents in other sites. Indeed, two population-based studies from Monrovia, Liberia during the Ebola epidemic showed that Liberians who trusted the government were more likely to take precautions against Ebola, be compliant with Ebola control policies and use health services [19,20]. Other studies have also shown that trusting government leadership is predictive of compliance to policies [21,22].

This study also showed that boys and girls across sites rely on different sources of information for COVID-19. Girls in some sites relied on getting information from their family members whereas boys in some sites got their information from health professionals or other websites on the internet. This created differences in the information they believed and their perceived severity. For example, girls believed in alternative natural remedies in preventing the virus which was usually communicated by their parents, teachers or neighbors. Boys, on the other hand, demonstrated an increased perceived severity and thought the 24-hour news medium was increasing their stress. In previous literature, sources of information for girls include parents and internet [23,24]. Burnout due to information overload from social media, specifically in relation to COVID-19, has also been documented within the literature but differences by sex have not been investigated [12-14].

The findings also showed that the type of information about COVID-19 from these sources of information were primarily about the virus itself, how it is transmitted, the symptoms, how it can be prevented, and the origin of the virus. Interestingly, across the sites, our findings show that adolescents follow a mental ranking to determine the trustworthiness of the sources, which was largely based on whether the information was logical and if it could be cross-verified. Previous research has showed that a source’s credibility is based on: who is the author or publisher; the author’s/publishers’ previous knowledge, experience, and writing style; whether diverse opinions are presented; the details offered by the source; and when the article was published [25,26]. Knowledge can be information and disinformation. We see there is a combination of both in almost all sites. This is in part by the ever-evolving evidence around COVID-19 preventative behaviors, uncertainty around the origin of the virus and perceived severity [11,27,28].

No study is without limitations. This study was conducted with a purposive sample of participants who were part of an existing cohort study. Therefore, there might have been some selection bias in terms of who volunteered to participate in the study and who did not, affecting generalizability. However, the fact that these FGDs were done across different countries and the results were similar suggests that there are commonalities in terms of how the adolescents are navigating the pandemic. Second, two sites (New Orleans and Flanders) found it easier to recruit girls in comparison to boys to be part of the study. Third, translation, transcription and back translation is based on the knowledge of researchers and interpreters. Poor implementation and documentation of back translation processes in many studies indicates that alternatives to back translation may be appropriate [29]. However, the facilitators and transcribers who conducted and translated these FGDs were trained beforehand and have conducted other FGDs for GEAS. Lastly, the data collection modalities were different between sites which could affect the type of information obtained.

Despite these limitations, this study has both research and programmatic implications. In terms of research, more research is needed to tease out whether adolescents’ who perceived their sources as more trustworthy actually have more accurate knowledge about COVID-19. Second, future research should look at the relationship between the number of trustworthy sources, perceived stress and COVID-19 preventative behaviors. In terms of programmatic implications: it is important that messaging from all trusted sources are accurate and tailored to the needs of adolescents’. Past studies have shown that using multiple social behavioral change communication mechanisms through different channels is most effective at creating sustained change [30]. While it is important that messaging from all trusted sources are accurate and tailored to the needs of adolescents, this study did show that boys and girls received information about COVID-19 differently. For girls, communication campaigns may want to design strategies that also aim at reaching family members and teachers, whereas for boys, health organizations and government sources may be the best channel for ensuring accurate information is received.

## Additional material


Online Supplementary Document
