# Supplementary material for: How do adolescents navigate COVID-19 information, and why does it matter?
Source: J Glob Health. 2021 Dec 25;11:03110. doi: 10.7189/jogh.11.03110 (PMC8709901; doi:10.7189/jogh.11.03110)
Supplement: Online Supplementary Document [file jogh-11-03110-s001.pdf]

Supplementary Document

Table S1. Sampling frame, sampling and recruitment and data collection modality

Site	Participant sampling frame	Participant Sampling and recruitment	Data collection modality	Data collection dates	Number of COVID cases in the site during data collection
Kinshasa, DRC	GEAS round 3 participants	Schools, community centers and phone	In-person at community centers	November 2020	Few or no cases
Shanghai, China	GEAS round 2 participants and adolescents from a nearby school	Schools	In-person at school	June 2020	Few or no cases
Semarang and Denpasar, Indonesia	GEAS round 1 participants	Schools	Online	August 2020	Many cases
New Orleans, USA	GEAS round 1 participants	Mass texting	Online	November 2020	Many cases
Flanders, Belgium	GEAS round 1 participants	Phone outreach via texting and phone	Online	September-October 2020	Some cases

GEAS – Global Early Adolescent Study

Table S2. Stratification of FGDs

Site	Stratification	Total number of FGDs	Number of participants
Kinshasa, DRC	Sex	2 FGDs with boys	14 boys 14 –18 years
		2 FGDs with girls	17 girls 14 –18 years
Shanghai, China	Sex	1 FGD with older boys	10 boys 16 –18 years
		1 FGD with younger boys	10 boys 14 – 15 years
	Age (14–15 years; 16–18 years)	1 FGD with older girls	10 girls 16 –18 years
		1 FGD with younger girls	10 girls 14 –15 years
Semarang and Denpasar, Indonesia	Sex	2 FGD with lower SES boys	8 boys lower SES in Denpasar 14-18 years
			6 boys lower SES in Semarang 14-18 years
		2 FGD with higher SES boys	8 boys higher SES in Denpasar 14–18 years
			7 boys higher SES in Semarang 14–18 years
	Socio-economic status (SES)	2 FGD with lower SES girls	8 girls lower SES Denpasar 14–18 years

	(based on wealth index during baseline: lower and higher)		8 girls lower SES in Semarang 14–18 years
		2 FGD with higher SES girls	8 girls higher SES Denpasar 14–18 years
			6 girls higher SES Semarang 14–18 years
New Orleans, USA	Sex	1 FGD with higher SES boys	6 boys higher SES 14–16 years
	SES (based on wealth index during baseline: lower and higher)	1 FGD with higher SES girls	4 girls higher SES 14–16 years
		2 FGDs with lower SES girls	12 girls lower SES 14–16 years
Flanders, Belgium	None	2 FGDs with boys and girls together	7 girls between 14 –16 years
			3 boys between 14 –15 years
**Total**		22 FGDs	161

SES – Socio-Economic Status, FGD – Focus Group Discussions

Table S3. Total number of codes by site

	**Total number of codes for the thematic group by site**				
**Thematic Code**	**Kinshasa, DRC**	**Shanghai, China**	**Semarang and Denpasar, Indonesia**	**New Orleans, USA**	**Flanders, Belgium**
Sources of COVID information_Trustworthy	9	25	115	3	7
Sources of COVID information_Trustworthy_How determined	11	14	113	5	15
Sources of COVID information_untrustworthy	3	9	77	5	2
Sources of COVID information_untrustworthy_how determined	2	8	62	2	3
Knowledge of COVID_beliefs/myths	8	18	13	1	2
Knowledge of COVID_description	10	15	18	5	5
Knowledge of COVID_prevention	27	122	156	15	10
Knowledge of COVID_spread	4	10	19	3	6
Knowledge of COVID_symptoms	7	15	28	4	2
Knowledge of COVID_transmission	17	39	109	5	3
Knowledge of COVID_treatment	2	1	2	0	3
Attitudes about the virus_changes in perceived severity	9	40	88	13	20
Attitudes about the virus_current perceived severity	15	35	136	8	5
Total number of codes	124	351	936	69	83
